# *N*-Glycan Profiles of Neuraminidase from Avian Influenza Viruses

**DOI:** 10.3390/v16020190

**Published:** 2024-01-26

**Authors:** Wentian Chen, Tianran Ma, Sinuo Liu, Yaogang Zhong, Hanjie Yu, Jian Shu, Xiurong Wang, Zheng Li

**Affiliations:** 1 Laboratory for Functional Glycomics, College of Life Sciences, Northwest University, Xi’an 710069, China; cwt@nwu.edu.cn (W.C.); shujian@nwu.edu.cn (J.S.); 2 National Key Laboratory of Veterinary Biotechnology, Harbin Veterinary Research Institute, Chinese Academy of Agricultural Science, Harbin 150001, China; wangxiurong@caas.cn

**Keywords:** avian influenza virus, neuraminidase, *N*-glycan profiles, lecin microarrays, MALDI-TOF/TOF-MS

## Abstract

The cleavage of sialic acids by neuraminidase (NA) facilitates the spread of influenza A virus (IV) descendants. Understanding the enzymatic activity of NA aids research into the transmission of IVs. An effective method for purifying NA was developed using *p*-aminophenyloxamic acid-modified functionalized hydroxylated magnetic particles (AAMPs), and from 0.299 to 0.401 mg of NA from eight IV strains was isolated by 1 mg AAMP. A combination of lectin microarrays and MALDI-TOF/TOF-MS was employed to investigate the *N*-glycans of isolated NAs. We found that more than 20 *N*-glycans were identified, and 16 glycan peaks were identical in the strains derived from chicken embryo cultivation. Multi-antennae, bisected, or core-fucosylated *N*-glycans are common in all the NAs. The terminal residues of *N*-glycans are predominantly composed of galactose and *N*-acetylglucosamine residues. Meanwhile, sialic acid residue was uncommon in these *N*-glycans. Further computational docking analysis predicted the interaction mechanism between NA and *p*-aminophenyloxamic acid.

## 1. Introduction

Influenza A viruses (IVs), which belong to the Orthomyxoviridae family, have has important effects throughout human history. Various subtypes, including human IVs, such as the H1N1, H2N2, and H3N2 subtypes, or highly pathogenic avian IVs, like the H5N1, H7N9, and H9N2 subtypes, are also responsible for millions of human deaths [1]. IV infections even affect subsequent SARS-CoV-2 infections in both cultured cells and mice [2]. Two envelope glycoproteins, HA (hemagglutinin) and NA (neuraminidase), play crucial roles in the invasion of host cells and the release of progeny virions, respectively [3].

Homotetrameric NA is a type II membrane protein. Nascent NA consists of four parts: the cytoplasm tail, transmembrane, stalk, and global domains [4]. While the crystal structures of the global domains have been elucidated, the other regions remain poorly understood. The different subtypes of NA are composed of 450–480 amino acids, displaying low-level sequence similarity, but conserved topologies: the six-bladed β-propeller fold constitutes an enzymatic activity domain [5]. As an exoglycosidase, the mechanism of SA (sialic acids, Neu5Ac) excision has been extensively described. The SA molecule adopts a chair conformation in the enzymatic center. After the formation of an oxocarbonium ion at the C2 atom of SA, strong ionic interactions occur between the carboxylate group of SA and the peripheral alkaline residues, leading to the subsequent cleavage of the glycosidic bond. Numerous basic and acid residues comprise the enzymatic center in NA from Type A and Type B IVs (e.g., Arg118, Asp151, Arg152, Arg224, Glu276, Arg292, and Arg371 in the N1 numbering system) [6,7]. Conserved Tyr406 is also a key catalytic residue that serves as a nucleophile [8].

Interestingly, numerous studies have found that *N*-glycosylation is crucial for the biological function of HA, while there is relatively little research on the glycosylation of NA. This may be because the isolation of NA poses certain challenges. Currently, four NA inhibitors are commonly licensed for the treatment and prevention of influenza: zanamivir, oseltamivir, peramivir, and laninamivir [9]. These inhibitors are analogs of the transition-state analog 2,3-dehydro-2-deoxy-*N*-acetylneuraminic acid substrate (DANA), and the substitution of different DANA groups can enhance their binding ability. Similarly, a series of analogs have been used for NA isolation based on the enzymatic center, such as MUNANA (4-methylumbelliferyl-α-d-*N*-acetylneuraminate) [10], octylglucoside (1-O-n-octyl-4-D-glucopyranoside) [11,12], and *p*-aminophenyl-2-acetamido-2-deoxy-1-thio-β-d-glucopyranoside [12].

In this study, we propose an efficient strategy for NA purification using *p*-aminophenyloxamic acid-modified hydroxyl-functionalized magnetic particles (AAMPs). A lectin microarray method combined with MALDI-TOF/TOF-MS analysis revealed distinct *N*-glycan profiles and relationships among eight strains of NAs. Computational docking analysis further elucidated the possible interaction conformation between NA and *p*-aminophenyloxamic acid.

## 2. Materials and Methods

### 2.1. Preparation of the AAMPs

Functionalized magnetic particles were widely used for various modes of glycoprotein or glycan-binding protein isolation in our previous works [13,14,15,16]. Briefly, *p*-aminophenyloxamic acid was immobilized on the hydroxyl-coated magnetic particles through several steps. Firstly, the epoxy-coated magnetic particles (200 mg, homemade) were washed with anhydrous ethanol and reacted with *p*-phenylenediamine (0.1081 g, dissolved in 50 mL anhydrous ethanol) in a stirrer at 45 °C and 500 rpm for 4 h. After that, the *p*-phenylenediamine-coated magnetic particles were washed with anhydrous ethanol and ultrapure water, followed by stirring with 0.126 g oxalic acid (dissolved in 50 mL 0.2 M MES buffer (2-(N-Morpholino)ethanesulfonic acid), pH = 5.0). Then, a catalytic amount of EDC (1-Ethyl-3-(3-dimethylaminopropyl) carbodiimide (0.1970 g), Sigma-Aldrich, St. Louis, MO, USA) and 0.1151 g NHS (N-Hydroxysuccinimide, Thermo Fisher, Waltham, MA, USA) were mixed in the reaction system at 4 °C and 500 rpm for 10 h. Finally, the AAMPs were separated using a magnetic separator and stored in ultrapure water at 4 °C.

### 2.2. Viral Whole Protein Preparation

Eight influenza strains, including H5N1DK (A/Duck/Guangdong/17/2008(H5N1)), H5N1CK (A/Chicken/Guangxi/4/2009(H5N1)), H5N2MD (A/Mallard/Jiangxi/16/2005(H5N2)), H5N2OT (A/Ostrich/Denmark/96-72420/1996(H5N2)), H7N1FL (A/Fowl/Rostock/45/1934(H7N1)), H7N2CK (A/Chicken/Hebei/1/2002(H7N2)), H9N2CK (A/Chicken/Fujian/S-1-521/2008(H9N1)), and H9N2DK (A/Duck/Guangdong/S-7-134/2004(H9N2)), were propagated in the chorioallantoic fluid of 10-day-old embryonated eggs at 37 °C. The virus titer of allantoic fluid was test by hemagglutination, and the viruses were collected by discontinuous sucrose density gradient centrifugation as previously described [17,18]. Viral whole proteins were extracted with a mixture of ether and ethanol and quantified by the Bradford method [19]. The protein of negative allantoic fluid was selected as a control.

### 2.3. NA Isolation

In each sample, 3 mg AAMP was initially blocked by 500 μL blocking buffer (0.1% octylpyranoid glucoside, 0.1 M sodium acetate, pH = 5.5) in a Vapour-bathing Constant Temperature Vibrator (ZWY-2101C, Shanghai Zhicheng Ltd., Shanghai, China) for 30 min at 37 °C. Following the removal of the supernatant using a magnetic separator, the AAMPs were shaken with 1 mL of balance buffer (0.1% octylpyranoid glucoside, 0.3 M sodium bicarbonate, pH = 9.1) and binding buffer (0.1% octylpyranoid glucoside, 0.1 M sodium acetate, pH = 5.5) in that order. Approximately 5 mg of viral whole protein was added to a 1 mL mixture of 2 × binding buffer and ultrapure water (1:1), labeled as the “original”. About 950 μL of the viral protein solution was incubated with the activated AAMPs for 2 h, after which the unbound proteins were separated using a magnetic separator (labeled as the “supernatant”) and removed by washing twice with 1 mL of washing buffer (0.15% octylpyranoid glucoside, 0.15 M sodium acetate, pH = 5.5). The waste fluid was labeled as “washing”. Finally, NAs were eluted using 500 μL of elution buffer for 2 h (0.2% octylpyranoid glucoside, 2 mM CaCl_2_, 0.1 M sodium bicarbonate, pH = 9.1). The supernatant was labeled as “elution”. The same steps were also applied to the negative allantoic fluid. All the above collections were used for further SDS-PAGE analysis [20,21].

### 2.4. NA Identification by MALDI-TOF-MS

MALDI-TOF-MS (Microflex MALDI-TOF, Bruker Daltonics) was utilized for NA identification. A total of 0.2 mg of isolated protein was concentrated and desalted using size-exclusion spin filtration (Amicon Ultra—0.5 10 kDa device, Millipore). Subsequently, the samples were washed twice with ultra-pure water at 4 °C and centrifuged at 14,000× *g* for 15 min. After centrifugation, the supernatants of the treated samples were collected and lyophilized using an Alpha 2–4 freeze dryer (Martin Christ, Germany). Similar procedures were used in keeping with the previous reports [22,23].

### 2.5. Glycopattern Analysis of NA Using Lectin Microarray

The extracted NAs were labeled with Cy3 fluorescent dye (GE Healthcare) and purified using Sephadex G-25 columns following the manufacturer’s instructions. The detailed design and fabrication of the lectin microarray have been described in previous reports [17,24]. Simply put, 4 μg of Cy5-labeled NA was diluted in 0.5 mL of incubation buffer and incubated with the homemade lectin microarrays for 3 h. Each slide was washed by 1 × PBST (Phosphate-Buffered Saline with Tween 20) and 1 × PBS (Phosphate-Buffered Saline) alternately. The microarray was scanned using a Genepix 4000 B confocal scanner (Axon Instruments, USA). The average background was subtracted, and values less than the average background ±2 SD (standard deviation) were removed from each data point. The fluorescence intensity of each spot was extracted using Gene Pix software (version 6.0; Axon Instruments Inc., Sunnyvale, CA, USA). The median of the effective data points of each lectin was globally normalized to the sum of the medians of all the effective data points for each lectin in one block. Each sample was consistently observed with three repeated slides. The normalized medians of each lectin from 9 repeated blocks were averaged, and the SD values were determined. The NFIs (Normalized Fluorescent Intensities) of the lectins from eight strains were compared based on the fold changes. The fold changes in pairs (with *p*-values lower than 0.05) with the NFIs of lectins were classified into three categories to evaluate whether the glycopatterns of NAs were altered between the two samples: (i) the results showing significant increases in NFIs (fold change 1.50, *p* < 0.05), (ii) the results showing significant decreases in NFIs (fold change 0.67, *p* < 0.05), and (iii) the results showing almost even NFIs (fold change ranges from 0.66 to 1.50, no significant difference). Differences between the two arbitrary datasets were tested with Paired student’s *t*-test using GraphPad Prism 7.0. The original data were further analyzed by Expander 6.0 software to perform HCE (hierarchical clustering analysis).

### 2.6. Characterization of N-Glycan Profiles of NAs by MALDI-TOF/TOF-MS

Similar to our previous reports [25,26], the *N*-glycans of the isolated NAs were released by PNGase F glycosidase. The purified *N*-glycans of NA were characterized by MALDI-TOF/TOF-MS (Ultra eXtreme, Bruker Daltonics; Bremen, Germany) as described previously. After desalting with Sepharose 4B hydrophilic resin (Sigma, USA), the glycan mixture was dissolved in 5 μL of water, and 1 μL was spotted on an MTP AnchorChip var/384 sample target (Bruker Daltonics, USA). Subsequently, an equal volume of 20 mg/mL 2,5-dihydroxybenzoic acid was spotted to recrystallize the glycans. A total of 1500 laser shots per pixel were collected, and the data were acquired using the Flex software suite (FlexControl 3.3 and FlexAnalysis 3.3). Representative MS spectra of *N*-glycans with signal-to-noise ratios >3 were chosen and annotated using GlycoWorkbench 2.1 software.

### 2.7. Docking Analysis for NA and p-Aminophenyloxamic Acid Interaction

To further investigate the potential interaction between NA and *p*-aminophenyloxamic acid, the three-dimensional structure of the NA receptor and a series of ligands were used for docking analysis. The NA structure was derived from an N1 subtype report (PDB ID:2HTY) and processed by removing water molecules, adding non-polar hydrogen atoms, and computing Gasteiger charges using Autodock Tools [27,28]. The structure file of *p*-aminophenyloxamic acid was constructed using PyMol 0.99 software. Additionally, the SA analogs, including DANA, oseltamivir, and zanamivir, were extracted from the reported NA-ligand crystal structures in 2HTW, 2HU0, and 2HTQ, respectively [28]. The 3D structures of monosaccharides, including SA, Man (Mannose), Glc (glucose), and Xyl (Xylose), were created using the SWEET-2 webtool [29]. Automated docking simulation was performed by using the AutoDock Vina program. A grid box with size of 30 × 30 × 30 Å was selected for protein–ligand docking analysis in the center of NA enzymatic active site. All other docking parameters were set to their default [30].

## 3. Results

### 3.1. NA Isolation by the AAMPs

**Figure 1:**
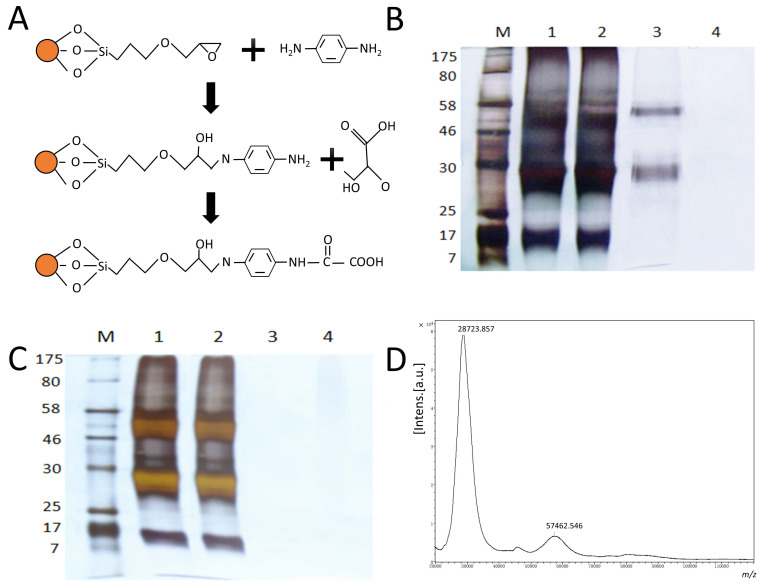
(**A**) The AAMPs were prepared through a two-step reaction. Firstly, the epoxy-coated Fe_3_O_4_ magnetic particles were modified by *p*-phenylenediamine, followed by coupling the oxalic acid. (**B**,**C**) Silver staining was performed for the SDS-PAGE analysis of isolated proteins from the infected chorioallantoic fluid and negative control. Line M: Marker; Line 1: Original; Line 2: Supernatant; Line 3: Elution; Line 4: Washing. (**D**) The MALDI-TOF-MS analysis of isolated proteins. The two prominent peaks at 28,723.857 and 57,462.546 Daltons indicate the isolated proteins belonging to NA.

The AAMPs were prepared by reacting an amino group of *p*-aminophenyloxamic acid with the epoxy-coated Fe_3_O_4_ magnetic particles (Figure 1A). The isolated procedures were optimized for the AAMP dosage, incubation time, and elution time. It was found that 3 mg of AAMP provided the most effective binding weight for the NAs, while an incubation time of 1.5 h and an elution time of 2 h provided an appropriate binding ability. The binding ability of *p*-aminophenyloxamic acid and NA varies with the pH, so we chose a binding and washing system at pH 5.5 and an elution system at pH 9.1 (Figure S1).

As depicted in Figure 1B, there are two distinct bands in the “elution” lines, with molecular weights of 28 KD and 58 KD, respectively. These bands differ from those in the “original” and “washing” lines. Notably, the molecular weight of the NA monomer is approximately 58 KD, while the 28 KD band is derived from the caducous NA global and stem domains [31]. In comparison, the elution line from the SDS-PAGE of uninfected allantoic fluid did not show any visible bands, indicating the high specificity of the AAMPs to NA. On the other hand, the MALDI-TOF-MS result identified two distinct peaks at 28,723.857 and 57,462.546 Daltons. These results confirm that AAMPs are a reliable tool for NA isolation.

NA was isolated from eight strains and quantified using the Bradford method. The results showed that 0.344 mg (H5N1CK), 0.315 mg (H5N1DK), 0.299 mg (H5N2MD), 0.307 mg (H5N2OT), 0.378 mg (H7N1FL), 0.345 mg (H7N2CK), 0.334 mg (H9N2CK), and 0.401 mg (H9N2DK) of NA proteins were isolated by 1 mg AAMP, while only 0.002 mg (Control) was detected in the uninfected allantoic fluid. It is evident that the amount of isolated NA ranged from 0.3 to 0.4 mg, with a consistent result across the different samples.

### 3.2. Glycopatterns Analysis of NAs by Lectin Microarray

**Figure 2:**
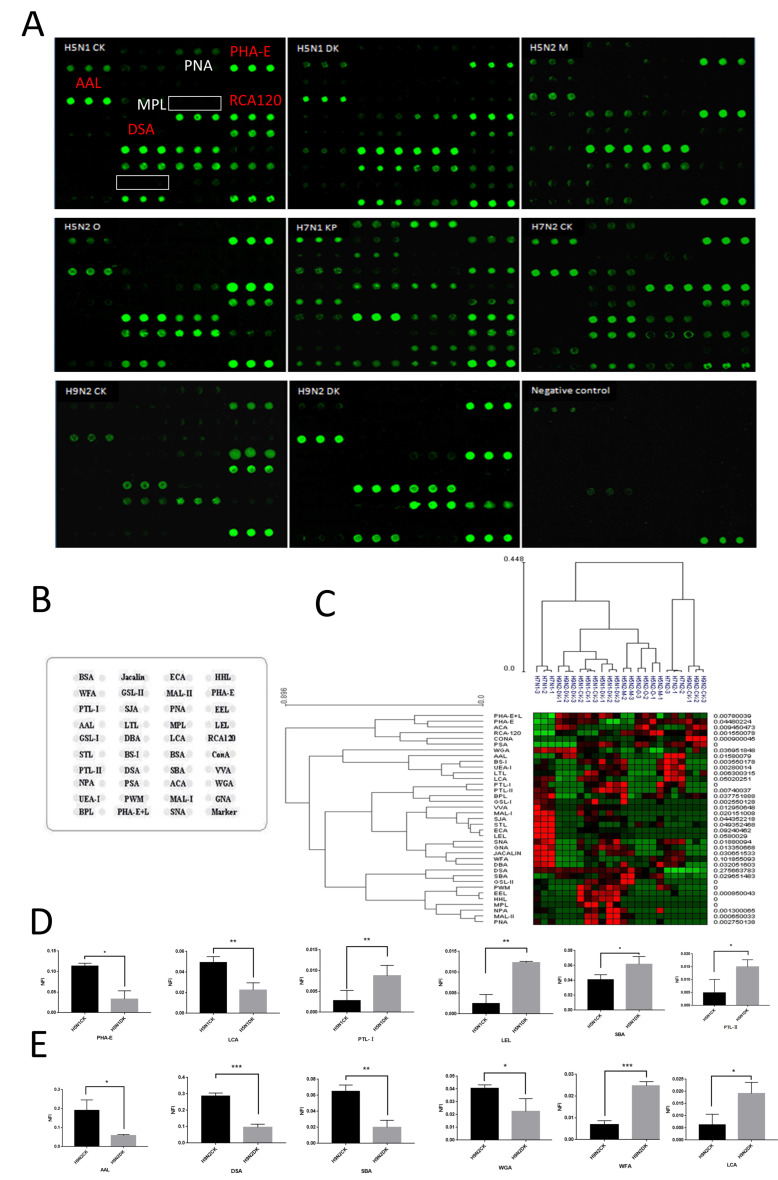
The lectin microarray analysis of NAs. (**A**) The lectin microarray has been incubated with NAs from eight strains and negative chicken embryo allantoic fluid. The lectins with affinity for all the NAs are labeled with red blocks, while the white blocks represent no affinity for all the NAs. (H5N1DK: A/Duck/Guangdong/17/2008(H5N1), H5N1CK: A/Chicken/Guangxi/4/2009(H5N1), H5N2MD: A/Mallard/Jiangxi/16/2005(H5N2), H5N2OT: A/Ostrich/Denmark/96-72420/1996(H5N2), H7N1FL: A/Fowl/Rostock/45/1934(H7N1), H7N2CK: A/Chicken/Hebei/1/2002(H7N2), H9N2CK: A/Chicken/Fujian/S-1-521/2008(H9N1), and H9N2DK: A/Duck/Guangdong/S-7-134/2004(H9N2). (**B**) The layout of lectin microarray. (**C**) HCE analysis of the *N*-glycan patterns of eight NAs. (**D**,**E**) Significance comparison of discrepant lectins in H5N1CK/H5N1DK and H9N2CK/H9N2DK groups (* *p* < 0.05, ** *p* < 0.01, and *** *p* < 0.001). Four lectins showed a decreasing trend of the NFIs (fold change < 0.67, *p* < 0.05) in some of the groups, with H5N1CK compared with H5N1DK, while four lectins showed an increasing trend of NFIs (fold change > 1.5, *p* < 0.05) in some of the groups, with H9N2CK compared with H9N2DK.

The lectin microarray provides an overall method used to identify the glycan structures. The layouts of the lectin microarray and NA-bound lectin microarrays are shown in Figure 2. The available NFIs (*N*-glycan fluorescence intensity) of each lectin were calculated and summarized in File S1. Although eight NAs showed different binding abilities to the lectins, compared to negative allantoic fluid, PHA-E (with affinity to bisecting GlcNAc, biantennary complex-type *N*-glycan with outer Gal), DSA (oligo GlcNAc), AAL (fucosylated glycans), and RCA120 (β-Gal, Galβ1-3GlcNAc) have a wide affinity for all the NAs. Meanwhile, GSL-I (αGalNAc, αGal, anti-A and B), PNA (Galβ1-3GalNAcα-Ser/Thr), MPL (Galβ1-3GalNAc), or PWM (Branched (LacNAc)n) gave no obvious signals of all the NAs.

The fold changes in the pairs with *p*-values lower than 0.05 and the NFIs of the lectins from two strains considering the different hosts are shown in File S1. For example, taking two original hosts from the H5N1 subtype, the lectin NFI H5N1CK/NFIH5N1DK with a ratio >1.5 or <0.66 (*p* < 0.05) was considered as showing differences. The results indicated that bisecting GlcNAc and biantennary *N*-glycans bind to PHA-E (fold change = 5.005, *p* = 0.003), while Fucα1-6GlcNAc (core fucose) binds to LCA (1.9743, *p* = 0.007) and shows increased NFIs in H5N1CK compared to those of H5N2DK. Additionally, Gal β-1,4GlcNAc (LacNAc) or poly LacNAc bind to LEL (0.2994, *p* = 0.015); terminal GalNAc, (GalNAc)n, and GalNAc α-1,3Gal bind to SBA (0.3282, *p* = 0.035); αGalNAc binds to PTL-Ⅰ (0.4702, *p* = 0.041); and Gal binds to PTL-Ⅱ (0.2743, *p* = 0.041). Interestingly, the comparison of the H9N2 subtypes also showed that LCA (2.2440, *p* = 0.024) and SBA (0.2334, *p* = 0.003) are distinctive in the H9N2CK/H9N2DK group. This may suggest that core fucosylation is abundant in the chicken host, while the GalNAc α-1,3Gal structure is relatively poor.

The heat map and HCA analyses of all the 37 lectins from the eight strains revealed that the lectins that bound to the glycans with acceptable reproducibility could reflect the glycopatterns from different strains. The glycopatterns varied greatly in the heat map, indicating significant differences between the strains. For instance, the glycopatterns of NAs from H9N2DK, H5N1DK, and H5N1CK or H5N2OT and H5N2MD exhibited relative similarities to each other compared to the other strains. This suggests that these strains may share similar glycosylation patterns, which could be useful for the future study and classification of influenza viruses.

### 3.3. N-Glycan Profiles of NAs by MALDI-TOF/TOF-MS Analysis

**Figure 3:**
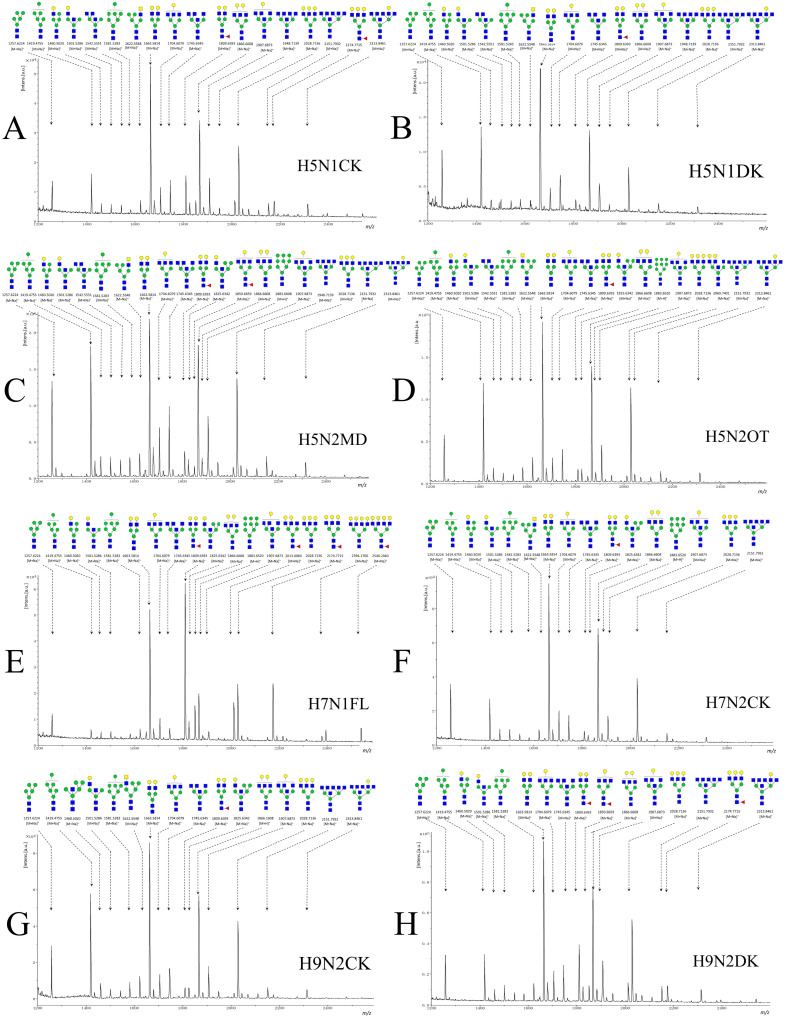
MALDI-TOF/TOF-MS spectra of the *N*-glycans from NAs from eight strains. Proposed structures and their *m*/*z* values are shown for each peak. Mannose (green circle), galactose (yellow circle), GalNAc (N-acetylgalcosamine, blue square), GlcNAc (N-acetylglucosamine, blue square), and fucose (red triangle) are shown. (**A**) H5N1CK; (**B**) H5N1DK; (**C**) H5N2MD; (**D**) H5N2OT; (**E**) H7N1FL; (**F**) H7N2CK; (**G**) H9N2CK; (**H**) H9N2DK.

To better understand the alterations in the glycan profiles of NAs, *N*-glycans were released from the isolated NAs using PNGase F and characterized using ultrafleXtreme MALDI-TOF/TOF-MS [32]. The MS spectra and proposed structures of the *N*-glycans are presented in Figure 3 and File S2. A total of 23 *N*-glycan peaks were identified and annotated, with from 19 to 21 *N*-glycans found in each NA sample. Most of the identified *N*-glycans were clustered between *m*/*z* 1200 and 2600. Among these, 16 *N*-glycans (*m*/*z* 1257.6224, 1419.4755, 1460.5020, 1501.5286, 1581.5283, 1622.5548, 1663.5814, 1704.6079, 1745.6345, 1809.6393, 1825.6342, 1850.6658, 1866.1608, 1907.6873, 2028.7136, and 2174.7714) presented in all the samples. The other *N*-glycans, such as the five-antenna *N*-glycans (*m*/*z* 2151.7932 and 2313.8460), were represented in most of the NA samples, while the four-antenna *N*-glycans (*m*/*z* 2394.1760 and 2540.2440) was only represented in H7N1FL. Among these overlapping *N*-glycans, the relative intensity (RI) of *N*-glycan at *m*/*z* 1663.5814 was highest; *m*/*z* 1866.1608 also resulted in relatively higher signals in parts of the NAs. The terminal residues of these *N*-glycans were predominantly galactose and GlcNAc, while sialic acid residue was uncommon (*N*-glycan nomenclature as described in Figure 3). These results indicate that the microheterogeneity of *N*-glycans is ubiquitous across the subtypes.

MALDI-TOF/TOF-MS/MS analysis was further performed to determine the exact glycan structures. As an example, the MALDI-TOF/TOF-MS/MS spectra of the precursor ions *m*/*z* 1622.5548, 1809.6393, 1907.6873, and 2151.7932 are also shown in Figure S2. Due to the low energy requirement, the major fragment ion B- and Y-type cleavages provided detailed sequencing information. The fragment ions that provide composition and linkage information were also detected. For example, the fragment ions Z4Z3 (881.303), Z4Z6 (1084.3803), Z5Z6 (1246.4332), and Z4 (1426.4965) in *m*/*z* 1809.6493 elucidated the occurrence of fucosylation. The fragment ions B3Y4 (1524.5446), B3 (1727.6239), and B4 (1948.803) elucidated the occurrence of five-antenna *N*-glycans.

### 3.4. Docking Analysis of NA and p-Aminophenyloxamic Acid

Docking analysis has been used to speculate on the potential binding mechanism between NA and different ligands (including *p*-aminophenyloxamic acid, SA analogs, and other contrasts). As a result, the docking results indicated all the candidates exhibited different abilities for NA binding. Notably, the binding energies of the other monosaccharaides were higher in comparison to those of the SA analogs; this reflects the weaker binding ability to NA. The binding energy of NA-*p*-aminophenyloxamic acid (−7.4 Kcal/mol) was similar to those of SA (−7.7 Kcal/mol), DANA (−7.7 Kcal/mol), zanamivir (−7.8 Kcal/mol), and oseltamivir (−7.6 Kcal/mol), but significantly lower than those of the contrasts, such as Man (−6.1 Kcal/mol), Glc (−5.4 Kcal/mol), and Xyl (−5.3 Kcal/mol).

**Figure 4:**
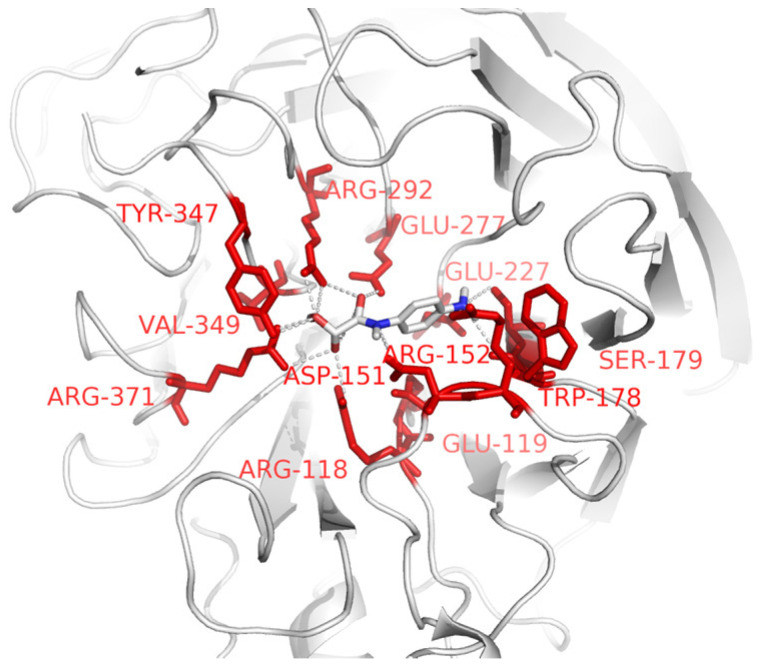
Potential interaction of NA (N1 subtype) and *p*-aminophenyloxamic acid. The residues which interact with the ligands are labeled. *p*-aminophenyloxamic acid embedded in the enzyme active center and formed numerous non-bond interactions with surrounding residues, especially the basic amino acids (Arg371, Arg292, Arg118, and Arg152), acidic amino acids (Glu119, Glu227, Glu277, and Asp151), and so on.

The conformation of docking analysis revealed that the tested ligands adopted similar conformations in the enzymatic center. The obtained docking results confirmed the capability of *p*-aminophenyloxamic acid in terms of effective molecular interaction with NA, when compared with that of the SA analogs. *p*-aminophenyloxamic acid embedded in the enzyme active center and formed numerous non-bond interactions with the surrounding residues, especially the basic amino acids (Arg371, Arg292, Arg118, and Arg152), acidic amino acids (Glu119, Glu227, Glu277, and Asp151), Trp178, Ser179, Tyr 347, and Val349 (Figure 4). More non-bond interactions between the SA analogs and NA can be observed in the other docking complexes. It is worth mentioning that there are no great differences in the coordinates of the NA active site compared to their original conformations in the PDB files. The highly conserved Asp151, Arg152, Trp178, Glu119, Arg118, Trp406, Arg371, Arg292, Glu276, and Arg224 residues play crucial roles in NA-ligand interactions. However, lesser non-bond interactions formed in the monosaccharide ligands like Mannose and Xylose (Figure S3).

## 4. Discussion and Conclusions

SA cleavage is a crucial step in the hydrolysis of oligosaccharide substrates by NA. However, the isolation and purification of NA remain challenging. As an exoglycosidase, NA binds and cleaves the SA residue. To address this issue, a series of SA analogs have been developed for anti-virus therapy and NA isolation based on substrate conformation, including DANA, zanamir, oseltamivir, 4-aminophenoxine (also known as para-aminobenzoic acid), and even *p*-aminophenyloxamic acid [32]. In this study, we aimed to isolate NA by coupling *p*-aminophenyloxamic acid to epoxy-coated magnetic particles. By optimizing the dosage, binding time, and elution time, the AAMPs were successfully applied to isolate NA from eight strains. The isolated proteins showed two bands in SDS-PAGE analysis, located at 57 KD and 28 KD, which corresponded to the two prominent peaks from MS analysis. We concluded that the 57 KD band was the glycosylated NA monomer, while the 28 KD band was the global domain of NA. In fact, the deciduous global domains of NA are common in the identified crystal structures. Based on docking analysis, the predicted binding energy of NA and *p*-aminophenyloxamic acid was slightly higher than those of the other SA analogs. We concluded that *p*-aminophenyloxamic acid adopted a lying conformation in the NA enzymatic center. The alkaline residues, especially Arg, play a crucial role in the binding of *p*-aminophenyloxamic acid. In summary, AAMPs provide an effective method for NA isolation.

HA and NA are a pair of glycoproteins that play an important role in IVs. The HA protein is responsible for the virus’s ability to invade the host cells, while the NA protein is responsible for releasing new viral particles. These proteins are regulated by various factors, with *N*-glycosylation being particularly noteworthy. The fickle distribution of *N*-glycosylation sites and various structures of *N*-glycan affect the functions of HA and NA in many ways. The combination of the lectin microarray and MALDI-TOF/TOF-MS analysis provides the comprehensive method for glycomic research. This approach has enabled the identification of over 20 *N*-glycans, with 16 of them being identical. Multi-antenna, bisected, or core-fucosylated *N*-glycans were found to be ubiquitous in all the NAs. The terminal residues of these *N*-glycans were predominantly galactose and GlcNAc, while the SA residue was uncommon. These characteristics are closely related to the chicken embryo cultivation process, indicating that the *N*-glycans of NA are derived from the host cell synthetic system and are consistent with the host cell surface [33]. A previous study also indicated as many as 14 *N*-glycosylation sites in both the N1 and N2 subtypes [34]. This means that at least 20 *N*-glycans (derived from five conserved *N*-glycosylation sites) surround the entire homotetramer. Despite the low-level homology between the different NA subtypes, the three-dimensional structures and distribution of *N*-glycosylation sites are conserved [35]. One *N*-glycosylation site, N146, is common in twelve NA subtypes and located at the top of the NA homotetramer. Four *N*-glycosylation sites, which are located at the stem domain, are also conserved in all the subtypes [36]. The *N*-glycosylation pattern is also different between the highly and lowly pathogenic avian influenza viruses. NAs from highly pathogenic avian IVs lack partial stem domains, which leads to the absence of 2–4 conserved *N*-glycosylation sites [37,38]. In addition, numerous unconserved *N*-glycosylation sites are distributed among the different subtypes, resulting in more complex glycosylation patterns. In conclusion, *N*-glycosylation is a complicated and dynamic process, and the microheterogeneity of the *N*-glycan profiles in these NAs is not amazing.

In conclusion, NA is a crucial protein in IV, but there has been a lack of glycobiology analysis. This research has developed a novel method for NA isolation using AAMPs. A lectin microarray and MALDI-TOF/TOF-MS analysis have revealed the microheterogeneity in the *N*-glycans of different NAs. The glycomics of different subtypes were discussed, providing an experimental foundation for viral surveillance and anti-IV therapy.

## Data Availability

Data are contained within the article and Supplementary Materials.

## Supplementary Materials

The following supporting information can be downloaded at: https://www.mdpi.com/article/10.3390/v16020190/s1, Figure S1. Optimization of isolated procedures has been discussed for the AAMP dosage (A), incubating time (B), and eluting time (C), according to Nano photometer detection. Figure S2. MALDI-TOF/TOF-MS analysis of the *N*-glycan precursor ion from MS spectra. Four *N*-glycan peaks, (A) *m*/*z* 1622.5548, (B) *m*/*z* 1809.6393, (C) *m*/*z* 1907.6873 and (D) *m*/*z* 2151.7932, subjected to MS/MS analysis. Mannose (green circle), galactose (yellow circle), GalNAc (N-acetylgalcosamine, blue square), GlcNAc (N-acetylglucosamine, blue square), and fucose (red triangle) are shown. Figure S3. Docking analysis of NA and other ligands. (A) *p*-aminophenyloxamic acid presented significant difference to parts of the monosaccharide ligands. Man: Mannose; Glc: glucose; Xyl: Xylose (B-H). The binding conformations between NA and SA, DANA, Zanamivir, Oseltamivir, Man, Glc, and Xyl were supported. The residues which interact with the ligands are labeled. File S1. The NFIs of lectin indicate the glycopattern in different NAs. Fold change in NAs H5N1CK/H5N1DK and H9N2CK/H9N2DK groups based on the ratio of the NFIs were also provided. File S2. *N*-glycan peaks of NAs showed the relative intensity in eight NAs. Mannose (green circle), galactose (yellow circle), GalNAc (N-acetylgalcosamine, blue square), GlcNAc (N-acetylglucosamine, blue square), and fucose (red triangle) are shown.
