# Supplementary material for: N-Glycan Profiles of Neuraminidase from Avian Influenza Viruses
Source: Viruses. 2024 Jan 26;16(2):190. doi: 10.3390/v16020190 (PMC10893399; doi:10.3390/v16020190)
Supplement: Supplementary file 1 [file viruses-16-00190-s001.zip › viruses-2793590-supplementary/supplementary file/File S1.pdf]

Lectin	Specificity	H5N1 CK	H5N1 DK	H5N2 M	H5N2 0	H7n2 W	H7N2 CK	H9N2 CK	H9N2 DK	H5N1 CK/H5N1DK	H9N2 CK/H9N2DK
JACALIN	Gal β1-3GalNAc α-Ser/Thr(T), GalNAcα-Ser/Thr(Tn)	0.0074 ± 0.0066	0.0094 ± 0.008	ND	0.0103 ± 0.0086	0.0273 ± 0.0031	0.0137 ± 0.002	0.0014 ± 0.0024	0.0003 ± 0.0005	0.7174	ND
ECA	Galβ-1,4GlcNAc	0.0157 ± 0.0013	0.01 ± 0.0008	ND	0.0045 ± 0.0031	0.088 ± 0.0078	0.0018 ± 0.0007	0.008 ± 0.0139	ND	1.5208	ND
HHL	PolyMan,(α1-3) and (α1-6) Man	0.007 ± 0.0047	0.0077 ± 0.001	ND	0.0005 ± 0.0009	0.0011 ± 0.0016	ND	0.0014 ± 0.0024	ND	0.7156	ND
WFA	GalNAcα/β1-3/6Gal	0.0232 ± 0.0067	0.0138 ± 0.001	0.0321 ± 0.014	0.024 ± 0.0054	0.0875 ± 0.0134	0.0511 ± 0.0079	0.0248 ± 0.0019	0.0068 ± 0.0018	1.8993	3.2453
GSL-II	αGalNAc, αGal	0.0102 ± 0.0072	0.0087 ± 0.0014	0.0057 ± 0.0099	0.0242 ± 0.0123	0.0034 ± 0.0031	ND	ND	ND	1.5163	ND
MAL-II	Sia2-3Galβ1-4Glc(NAc)	0.0047 ± 0.0035	0.0072 ± 0.0064	ND	0.0016 ± 0.0014	0.0003 ± 0.0003	ND	ND	ND	0.6911	ND
PHA-E	Bisecting GlcNAc, biantennary N-glycans	0.1133 ± 0.0069	0.0333 ± 0.0197	0.1262 ± 0.032	0.0675 ± 0.0292	0.0336 ± 0.0105	0.1162 ± 0.0061	0.119 ± 0.0244	0.1078 ± 0.0157	5.0050	1.1281
PTL-I	αGalNAc	0.0028 ± 0.0024	0.0088 ± 0.0025	ND	0.0083 ± 0.0072	0.0038 ± 0.0035	0.0055 ± 0.0024	ND	ND	0.4703	ND
SJA	Terminal GalNAc and Gal	0.0064 ± 0.0056	0.0075 ± 0.0014	ND	0.0047 ± 0.004	0.0305 ± 0.0122	0.001 ± 0.0017	ND	ND	1.1317	ND
PNA	Gal β1-3GalNAcα-Ser/Thr(T)	0.0048 ± 0.0042	0.0069 ± 0.004	ND	0.0025 ± 0.0019	0.002 ± 0.0017	0.0009 ± 0.0016	0.0019 ± 0.0033	ND	0.7844	ND
EEL	Galα1-3(Fucα1-2)Gal	0.0099 ± 0.0053	0.0093 ± 0.0012	ND	0.0016 ± 0.0018	0.0015 ± 0.0019	0.001 ± 0.0017	0.0015 ± 0.0026	ND	1.0526	ND
AAL	Fucα-1,6GlcNAc(core fucose),Fucα-1,3Galβ-1,4GlcNAc	0.088 ± 0.0183	0.1122 ± 0.0349	0.0519 ± 0.0096	0.0342 ± 0.0053	0.0154 ± 0.0014	0.2725 ± 0.0039	0.0579 ± 0.0055	0.1909 ± 0.0544	0.7447	0.2668
LTL	Fuc α1-3(Gal β1-4)GlcNAc, anti-H blood group specificity	0.0118 ± 0.0079	0.0089 ± 0.0019	0.0037 ± 0.0064	0.0052 ± 0.0045	0.0062 ± 0.0002	0.0177 ± 0.005	0.006 ± 0.0051	0.003 ± 0.0053	1.6452	ND
MPL	Gal β1-3GalNAc, GalNAc	0.0052	0.0032	ND	ND	0.0003	ND	ND	ND	4.4357	ND

		± 0.0024	± 0.0045			± 0.0005					
LEL	(GlcNAc)n,high mannose-type N-glycans	0.0025 ± 0.0022	0.0123 ± 0.0003	ND	0.0028 ± 0.0022	0.0602 ± 0.0044	ND	0.0018 ± 0.0031	ND	0.2944	ND
GSL-I	GlcNAc	0.0038 ± 0.004	ND	ND	0.0045 ± 0.0066	0.0037 ± 0.0011	ND	ND	ND	ND	ND
DBA	αGalNAc,Tn antigen, GalNAc α1-3((Fuc α1-2))Gal	0.0045 ± 0.0041	0.0077 ± 0.0048	0.0078 ± 0.0036	0.0205 ± 0.0124	0.0285 ± 0.0034	0.0158 ± 0.003	0.0008 ± 0.0014	ND	0.7512	ND
LCA	αMan,Fucα1-6GlcNAc(core fucose)	0.0492 ± 0.0057	0.0226 ± 0.0069	0.0151 ± 0.0013	0.0117 ± 0.0031	0.0471 ± 0.0061	0.0744 ± 0.008	0.0191 ± 0.0045	0.0062 ± 0.0044	1.9744	2.2440
RCA-120	βGal, Galβ-1, 4GlcNAc (type II), Galβ1-3GlcNAc (type I)	0.0443 ± 0.0051	0.1005 ± 0.0241	0.1617 ± 0.0445	0.1913 ± 0.0532	0.0024 ± 0.0027	0.1116 ± 0.0022	0.1422 ± 0.031	0.1026 ± 0.0089	0.4795	1.3436
STL	trimers and tetramers of GlcNAc, core (GlcNAc) of N-glycan, oligosaccharide containing GlcNAc and MurNAc	0.0066 ± 0.0058	ND	0.01 ± 0.0057	0.0086 ± 0.0087	0.0458 ± 0.0033	0.0003 ± 0.0005	0.0051 ± 0.0032	0.0023 ± 0.0012	ND	1.7649
BS-I	αGal and αGalNAc	0.0077 ± 0.0014	0.0095 ± 0.0051	ND	0.0073 ± 0.0065	0.0043 ± 0.0021	0.0128 ± 0.0046	ND	ND	0.7569	ND
CONA	High-Mannose, Man α1-6(Man α1-3)Man, terminal GlcNAc	0.0427 ± 0.0071	0.0236 ± 0.0069	0.0565 ± 0.0079	0.0125 ± 0.0016	0.0065 ± 0.0083	0.0371 ± 0.0046	0.2309 ± 0.0268	ND	1.6523	ND
PTL-II	Gal	0.0049 ± 0.0052	0.015 ± 0.0027	ND	0.0084 ± 0.0074	0.0088 ± 0.0014	0.0049 ± 0.0039	0.0023 ± 0.004	ND	0.2744	ND
DSA	B-D-GlcNAc, (GlcNAc β1-4)n, Gal β1-4GlcNAc	0.2783 ± 0.014	0.2833 ± 0.0184	0.2226 ± 0.0364	0.2824 ± 0.081	0.2873 ± 0.0129	0.073 ± 0.0093	0.0956 ± 0.0192	0.2868 ± 0.0177	0.9543	0.2994
SBA	Terminal GalNAc	0.0408 ± 0.0068	0.0618 ± 0.0102	0.0605 ± 0.0099	0.1308 ± 0.043	0.0199 ± 0.0089	0.0171 ± 0.003	0.02 ± 0.0087	0.065 ± 0.0077	0.6102	0.2334
VVA	Terminal GalNAc and GalNAcα-Ser/Thr(Tn)	0.0099 ± 0.0047	0.0077 ± 0.0017	ND	0.0005 ± 0.0009	0.0318 ± 0.0429	ND	0.0054 ± 0.0094	ND	1.1359	ND
NPA	High-Mannose, α1-6Man	0.0028 ± 0.0026	0.0084 ± 0.005	ND	0.0043 ± 0.004	0.0022 ± 0.0008	ND	ND	ND	0.3282	ND
PSA	Fucose α1-6GlcNAc(core fucose)	0.0385 ± 0.001	0.0486 ±	0.044 ± 0.0159	0.0199 ±	ND	0.0455 ±	0.0513 ±	0.0043 ±	0.7886	ND

			0.0135		0.0034		0.0059	0.0156	0.0074		
ACA	Gal β1-3GalNAc α-Ser/Thr(T)	0.0403 ± 0.0054	0.0281 ± 0.0035	0.1319 ± 0.0509	0.0359 ± 0.0043	0.007 ± 0.0024	0.0219 ± 0.0066	0.1294 ± 0.0297	0.1327 ± 0.0209	1.4686	0.9775
WGA	Terminal GlcNAc, (GlcNAc)n	0.0241 ± 0.0021	0.0162 ± 0.0034	0.0145 ± 0.0077	0.0101 ± 0.0029	0.0381 ± 0.0041	0.0214 ± 0.0048	0.0224 ± 0.01	0.0404 ± 0.0028	1.2821	0.4899
UEA-I	Fucα1-2Galβ1-4Glc(NAc)	0.003 ± 0.0026	0.0091 ± 0.0019	ND	0.0084 ± 0.0073	0.0059 ± 0.0027	0.0207 ± 0.0058	ND	ND	0.4327	ND
PWM	GlcNAc,Branched (LacNAc)n	0.0092 ± 0.0066	0.0089 ± 0.0021	ND	0.0063 ± 0.0041	0.002 ± 0.0018	0.0029 ± 0.0032	ND	ND	0.9807	ND
MAL-I	Galβ-1,4GlcNAc	0.0109 ± 0.0049	0.0053 ± 0.0072	ND	0.0001 ± 0.0002	0.0256 ± 0.0049	ND	ND	ND	5.4379	ND
GNA	Terminal α1-3Man	0.0067 ± 0.0029	0.0129 ± 0.0019	ND	0.0058 ± 0.0022	0.0162 ± 0.0024	0.0044 ± 0.0021	0.0028 ± 0.0049	ND	0.6147	ND
BPL	Galβ1-3GalNAc,Terminal GalNAc	0.0072 ± 0.0045	0.0112 ± 0.0032	0.0116 ± 0.0051	0.0192 ± 0.0179	0.0261 ± 0.0101	0.0072 ± 0.0042	0.0149 ± 0.0021	0.0016 ± 0.0027	0.7054	ND
PHA-E+L	Tri- and tetra-antennary complex-type N-glycan	0.046 ± 0.0076	0.0479 ± 0.011	0.044 ± 0.0071	0.0159 ± 0.0027	0.0143 ± 0.0056	0.0343 ± 0.0109	0.0321 ± 0.0101	0.0492 ± 0.0085	0.9447	0.5690
SNA	Sia2-6Galβ1-4Glc(NAc)	0.0056 ± 0.0044	0.0127 ± 0.0049	ND	0.0037 ± 0.0036	0.0153 ± 0.0043	0.0133 ± 0.0013	0.0019 ± 0.003	ND	0.2600	ND
