# Supplementary material for: N-Glycan Profiles of Neuraminidase from Avian Influenza Viruses
Source: Viruses. 2024 Jan 26;16(2):190. doi: 10.3390/v16020190 (PMC10893399; doi:10.3390/v16020190)
Supplement: Supplementary file 1 [file viruses-16-00190-s001.zip › viruses-2793590-supplementary/supplementary file/File S2-new.pdf]

No.	Calculated *m/z*	Ion type	Proposed *N*-glycan structure	Relative intensities of *N*-glycans *							
				H5N1CK	H5N1DK	H5N2MD	H5N2OT	H7N1FL	H7N2CK	H9N2CK	H9N2DK
1	1257.4226	Na^+	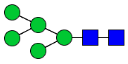	0.0475	0.0688	0.0523	0.0509	0.0425	0.0819	0.0777	0.0528
2	1419.4754	Na^+	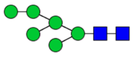	0.0530	0.1113	0.1148	0.1382	0.0291	0.0718	0.1575	0.0622
3	1460.5020	Na^+	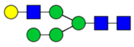	0.0161	0.0267	0.0247	0.0203	0.0247	0.0228	0.0204	0.0188
4	1501.5285	Na^+	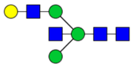	0.0159	0.0294	0.0194	0.0132	0.0261	0.0221	0.0107	0.0215
5	1542.5551	Na^+	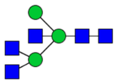	0.0154	0.0294	0.0168	0.0122	ND	0.0146	0.0107	0.0146
6	1581.5282	Na^+	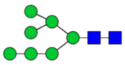	0.0095	0.0334	0.0155	0.0205	0.0196	0.0150	0.0212	0.0232
7	1622.5548	Na^+	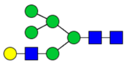	0.0290	0.0311	0.0266	0.0349	0.0335	0.0235	0.0340	0.0252
8	1663.5813	Na^+	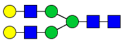	0.2218	0.2084	0.1961	0.2181	0.1791	0.2442	0.2264	0.1985
9	1704.6079	Na^+	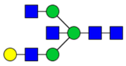	0.0452	0.0437	0.0544	0.0329	0.0352	0.0493	0.0321	0.0415
10	1745.6344	Na^+	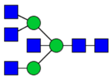	0.0525	0.0634	0.0758	0.0433	0.0228	0.0399	0.0386	0.0430
11	1809.6393	Na^+	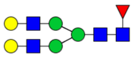	0.0650	0.0399	0.0277	0.0187	0.2106	0.0212	0.0135	0.0716
12	1825.6342	Na^+	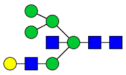	0.0265	0.0169	0.0182	0.0172	0.0336	0.0152	0.0135	0.0220
13	1850.6658	Na^+	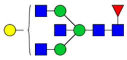	0.0288	0.0000	0.0115	0.0082	0.0519	0.0108	0.0000	0.0223
14	1866.6607	Na^+	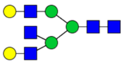	0.1367	0.1175	0.1389	0.1497	0.0682	0.1736	0.1499	0.1400
15	1883.6519	H^+	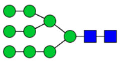	ND	ND	0.0208	0.0152	0.0147	0.0185	0.0072	0.0203
16	1907.6873	Na^+	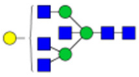	0.0532	0.0541	0.0241	0.0479	0.0195	0.0385	0.0410	0.0483

17	1948.7138	Na^+	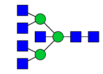	0.0095	0.0145	0.0155	0.0101	ND	0.0126	0.0091	0.0136
18	2028.7135	Na^+	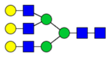	0.0992	0.0736	0.1037	0.1174	0.0765	0.0973	0.1108	0.1004
19	2151.7932	Na^+	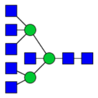	0.0251	0.0221	0.0216	0.0131	ND	0.0170	0.0152	0.0214
20	2174.7714	Na^+	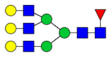	0.0283	0.0000	0.0081	0.0069	0.0739	0.0000	0.0000	0.0226
21	2313.8460	Na^+	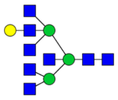	0.0217	0.0157	0.0136	0.0111	ND	0.0103	0.0105	0.0162
22	2394.1760	Na^+	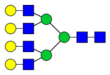	ND	ND	ND	ND	0.0175	ND	ND	ND
24	2540.2440	Na^+	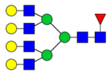	ND	ND	ND	ND	0.0210	ND	ND	ND

* **H5N1DK: A/Duck/Guangdong/17/2008(H5N1), H5N1CK: A/Chicken/Guangxi/4/2009(H5N1), H5N2MD: A/Mallard/Jiangxi/16/2005(H5N2), H5N2OT: A/Ostrich/Denmark/96-72420/1996(H5N2), H7N1FL: A/Fowl/Rostock/45/1934(H7N1), H7N2CK: A/Chicken/Hebei/1/2002(H7N2), H9N2CK: A/Chicken/Fujian/S-1-521/2008(H9N1) and H9N2DK: A/Duck/Guangdong/S-7-134/2004(H9N2).**
