# Supplementary figures and images for: N-Glycan Profiles of Neuraminidase from Avian Influenza Viruses
Source: Viruses. 2024 Jan 26;16(2):190. doi: 10.3390/v16020190 (PMC10893399; doi:10.3390/v16020190)

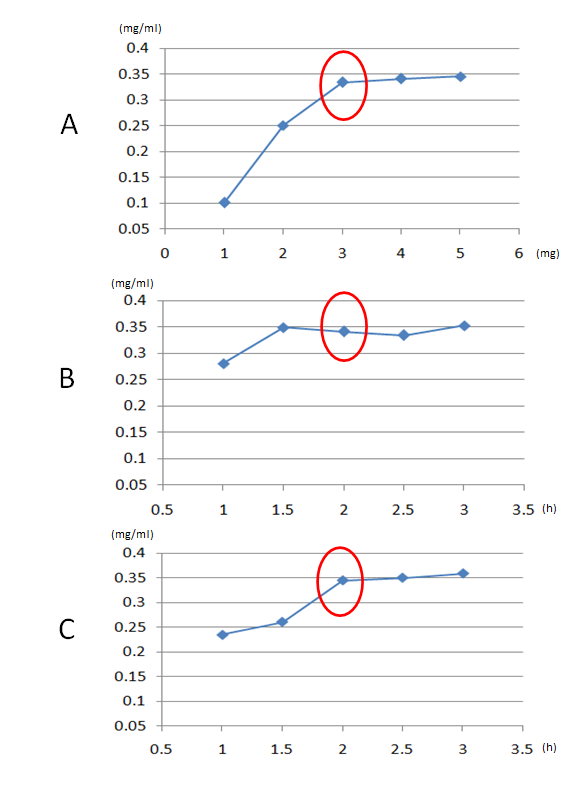

Supplement: Supplementary file 1 [file viruses-16-00190-s001.zip › viruses-2793590-supplementary/supplementary file/Figure S1.tif]

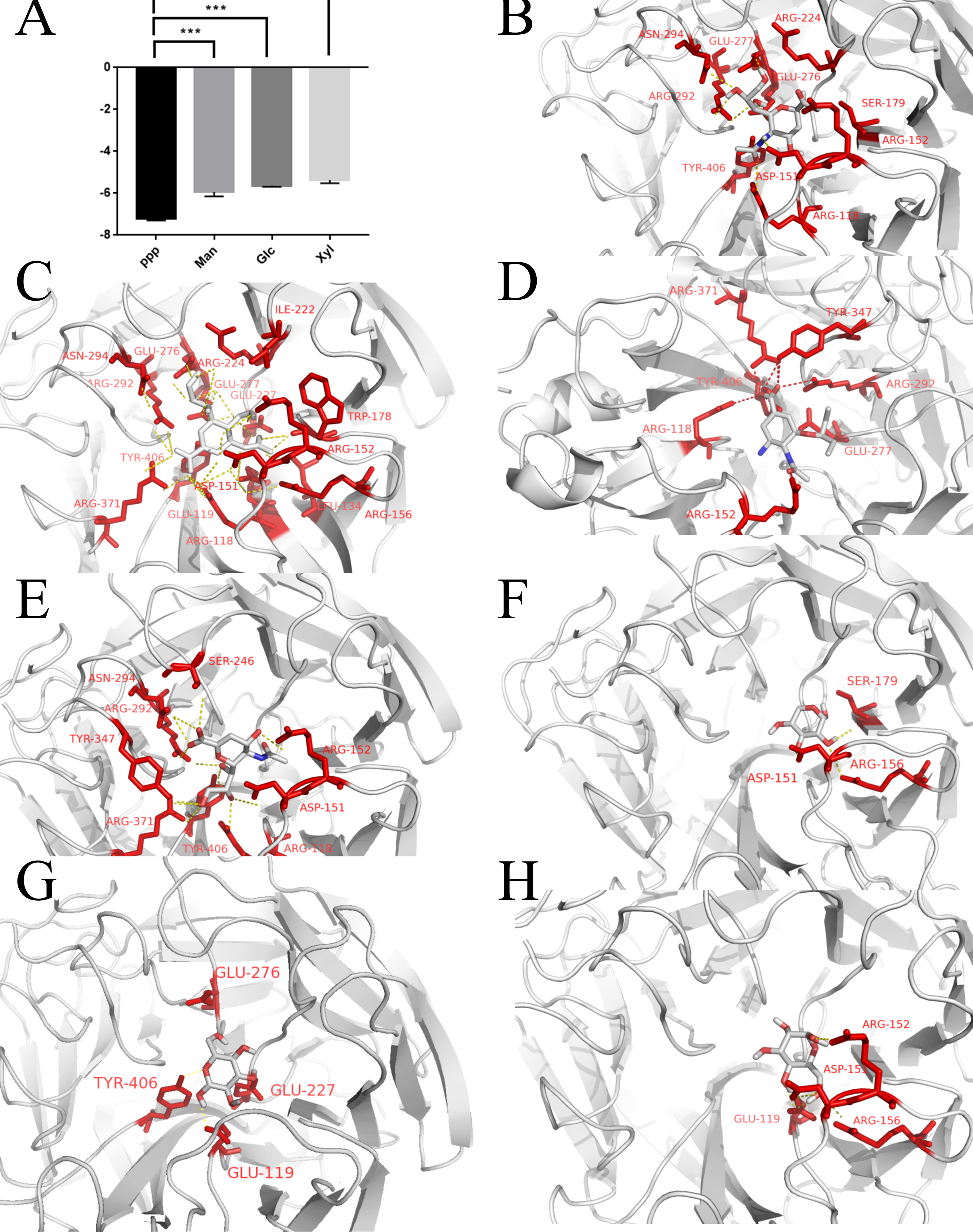

Supplement: Supplementary file 1 [file viruses-16-00190-s001.zip › viruses-2793590-supplementary/supplementary file/Figure S3-new.tif]
